# Xanthoma Disseminatum In a Pair of Blind, Deaf Male Twins

**DOI:** 10.5402/2011/342909

**Published:** 2011-03-15

**Authors:** Naveed Natanzi, David Peng, Eli Ahdoot, Sandra Ghatan, Amy Reinstandler, Ramin Ram

**Affiliations:** ^1^Western University of Health Sciences, 309 East Second Street, Pomona, CA 91766, USA; ^2^Department of Dermatology, University of Souther California, Los Angeles, CA 90033, USA; ^3^University of California, Los Angeles, CA 90095, USA

## Abstract

Xanthoma disseminatum (XD) is a rare normolipemic histiocytic disorder of non-Langerhans cell origin characterized by erythematous to tan/brown papules in flexor surfaces. Considered a generally benign, chronic disease of unknown etiology, XD typically affects the skin, mucous membranes, and less commonly, other organs. To date, there has been no typical or consistent inheritance pattern described, nor has it ever been considered as a component of any known syndrome. We describe, for the first time, two cases of XD in a pair of blind and deaf twin brothers.

## 1. Introduction

Xanthoma disseminatum (XD) is a disorder of non-Langerhans cell origin that is characterized by numerous, widely distributed red-yellow to red-brown xanthomatous deposits affecting the skin and mucosa in the absence of hyperlipidemia [1]. Although the natural history of the disease itself is usually benign, lesions in critical anatomical locations may result in significant morbidity and mortality [2, 3]. XD characteristically involves the flexural regions of the skin and mucous membranes; typically, the upper aerodigestive mucusa is involved [4, 5]. Systemic involvement is not uncommon, and lesions have been described in almost every organ system in the body [1, 6]. Among them, skeletal, bone marrow, hepatic, respiratory, ocular, and central nervous system involvement are most common [7].

As an extremely rare condition, XD has only been reported in roughly 100 patients, since it was first defined by Montgomery and Osterberg in 1938 [8]. It can occur in either sex and at any age, but it most commonly affects male children and young male adults [5, 9]. No typical inheritance pattern has previously been described, [10] nor has XD ever been associated with any other conditions as part of a genetic syndrome. We report, for the first time, two cases of XD in a pair of twin brothers, who developed blindness and deafness in the first decade of life.

## 2. Case Report

The patients are twin males who were born after a normal pregnancy with no known maternal exposure to drugs or viral infection. The authors do not declare whether the twins are genetically identical or not. Their medical histories and presentations were nearly identical and were significant for idiopathic, gradual loss of vision and hearing during early childhood. Both brothers developed complete blindness and deafness within the first decade of life. The brothers' two sisters and their children are free of both cutaneous and audiovisual abnormalities.

**Figure 1:**
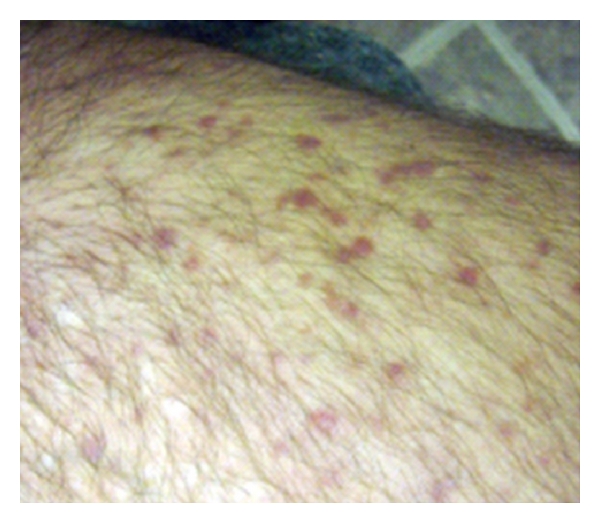
Lesions of xanthoma disseminatum on the leg revealing characteristic red-brown waxy lesions.

On examination, both patients had multiple facial lesions characterized by smooth, gelatinous-appearing papules, some of which coalesced into large plaques. There were also numerous small erythematous papules on the buttocks regions. The upper extremities were uninvolved, but the lower extremities had scattered well-defined red or red-brown papules that were waxy and densely clustered with coalescing patches (Figure 1). There were also areas of mottled hypopigmentation bilaterally on both patients' lower extremities, particularly focused in the knee and popliteal regions. 

On review of systems, the patients denied arthritis, fatigue, weight loss, abdominal pain, bone pain, shortness of breath, fevers, or chills. 

**Figure 2:**
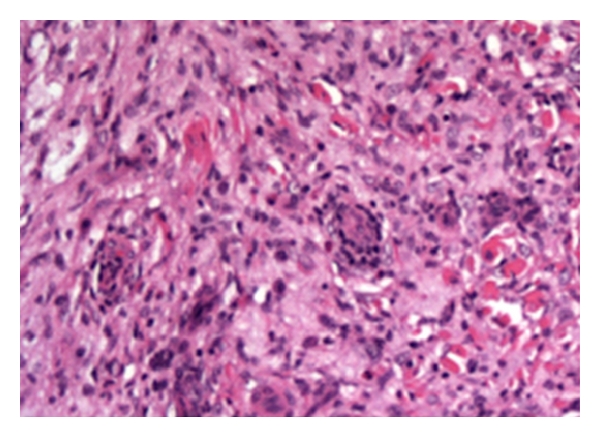
A biopsy section of the skin showing a diffuse histiocytic infiltrate with foamy cytoplasm and Touton giant cells.

A biopsy of each patient's knees showed a histiocytic cellular proliferation with plasma cells and multinucleated giant cells (Figure 2). The differential diagnosis included both reactive and nonreactive causes of histiocytoses. Reactive causes considered were lesions secondary to leishmaniasis, syphilis, or atypical mycobacterial infection. Nonreactive causes included Rasai-Dorfman disease, xanthoma disseminatum, or Langerhans cell histiocytosis. 

Doxycycline was empirically started at 100 mg twice a day to cover an atypical mycobacterial infection. Interestingly, one twin patient developed a self-limited gyrate erythema over his right flank only two weeks after starting therapy. During the course of his antibiotic treatment, it appeared that established lesions improved; however, numerous new lesions cropped up on his flanks and dorsal feet in a symmetric distribution. After 6 weeks of therapy, the patient discontinued the doxycycline. 

Complete blood count, basic metabolic panel, and thyroid and liver function tests were within normal limits. Lipid panel revealed low serum triglycerides, normal cholesterol levels and LDL with low HDL. Antinuclear antibodies (ANA) and rapid plasma regain (RPR) were negative. Urine histoplasma antigen was also negative. 

**Figure 3:**
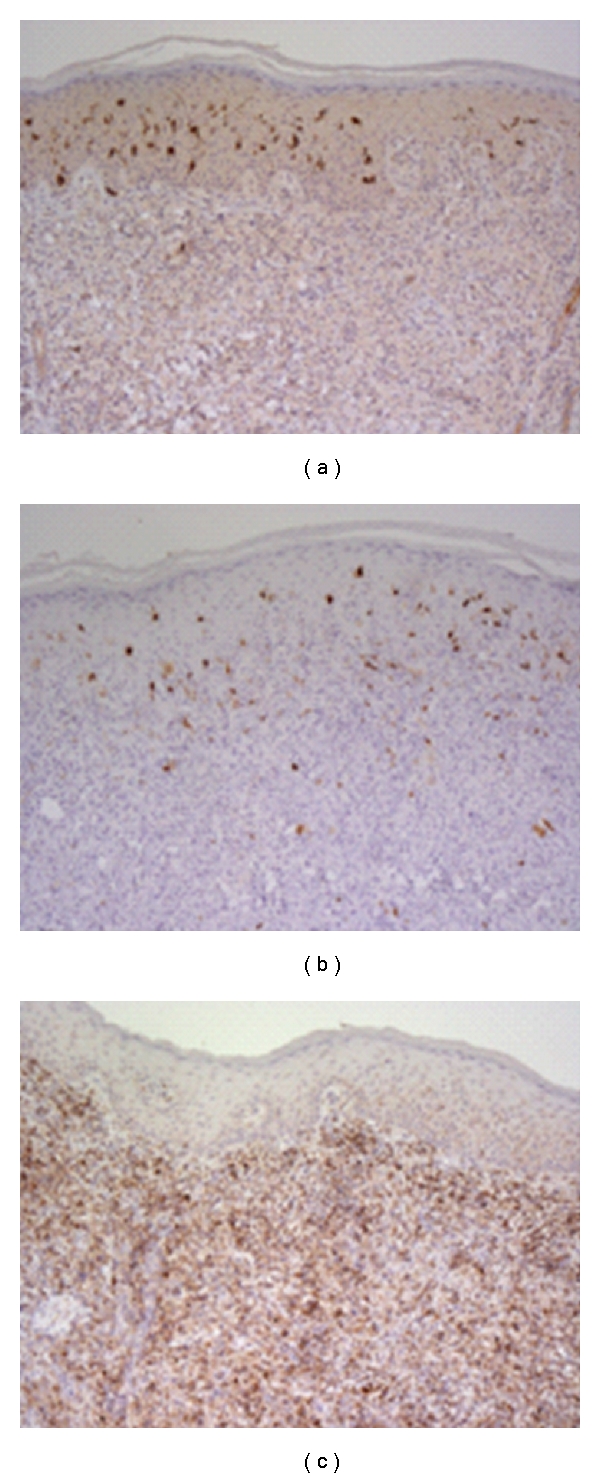
(a) Immunohistochemical labeling of the biopsy showing CD1a negativity. (b) Immunohistochemical labeling of the biopsy showing S100 negativity. (c) Immunohistochemical labeling of the biopsy CD68 positivity.

Immunohistochemical studies were performed to characterize the nature of the inflammatory infiltrate (Figure 3). The CD45, S-100, and CD1a stains were all negative, indicating that the cells were neither lymphocytes nor Langerhans cells. The CD68 stain was strongly positive, revealing the presence of numerous macrophages. The fascin stain was also positive, confirming that the cells were mature dendritic cells. These studies, in conjunction with the clinical presentation, favored a diagnosis of xanthoma disseminatum, and subsequently both twins were started on a trial of methotrexate therapy at 15 mg/wk. 

To look for systemic involvement, several CT scans with contrast were performed. The patients were found to have a normal head, neck, heart, lungs, gallbladder, liver, spleen, pancreas, adrenal glands, and kidneys. No retroperitoneal lymphadenopathy was demonstrated.

## 3. Discussion

Xanthoma disseminatum is a rare xanthomatosis belonging to a group of conditions known as the non-X histiocytoses. It is characterized by xanthomatous deposits throughout the body, particularly the flexural areas of the skin. Involvement is usually widespread and may become disfiguring. In contrast to other xanthomatoses, it is classically normolipemic [10, 11].

Xanthoma disseminatum was first described in 1869 by Von Grafe [12] but was not classified as a distinct entity until 1938 by Montgomery and Osterberg [8]. It was originally thought to overlap with other Langerhans cell histiocytoses, such as Hand-Schuller-Christian disease [2, 13]. However, advanced immunochemical staining techniques have since shown that it is clearly distinct from the Langerhans cell histiopathies. The histological demonstration of CD1a, S100, and Birbeck granules that is seen with Langherhans cell histiopathies is not seen in xanthoma disseminatum, [14–16] which instead expresses CD68 positivity and Touton giant cells, representing an exaggerated xanthomatoid reaction [17]. 

The diagnosis of xanthoma disseminatum is based on both clinical and pathological evidence. Clinical manifestations involving the skin, CNS, and respiratory tract are consistent with the diagnosis, which can be confirmed by biopsy with immunophenotyping and ultrastructural studies [18].

Although the disease is generally benign, there have been complications associated with the anatomical locations of the lesions. Xanthomatous lesions may not all be readily visible to the naked eye. Involvement of affected organs can be demonstrated by imaging via X-ray, CT, MRI, ultrasound, or ERCP. Approximately 40% of patients have mucocutaneous involvement of the upper digestive and respiratory tracts, resulting in hoarseness, dyspnea, and dysphagia [19]. This can cause life-threatening obstruction and asphyxiation, requiring tracheostomy [5, 19–22]. Extensive meningeal involvement in the region of the sella and hypophysis is thought to be the cause of the frequent association with diabetes insipidus [3, 23–25]. Meningeal involvement may also lead to seizures, ataxia, ophthalmoplegia, and growth retardation [20, 26–29]. 

There have been few advancements made in the development of a successful therapy for XD over the last decade. Most popular methods of treatment include a combination of chemotherapy, Clofibrate, radiotherapy, surgery, and steroid treatment [18]. Early treatment of aggressive subtypes of XD with chemotherapy drugs such as Cyclophosphamide, have notably better prognoses [30].

In our case, because no disease infiltration was seen on CT, it is unlikely that this was the cause of the patients' blindness and deafness. It is striking, however, that the brothers share both cutaneous and audiovisual symptoms, suggesting an association between the two.

Given the rarity of xanthoma disseminatum, with just over 100 cases reported in the literature, the majority of what is known about the disease and its pathogenesis comes from case reports. No typical inheritance pattern has previously been described in these reports nor has it ever been described as part of a genetic syndrome. This case of blind, deaf twin brothers, both with xanthoma disseminatum, is the first of its kind and suggests that there may be genetic component to this condition. Furthermore, these cases suggest that in some instances, XD might be associated with loss of hearing and vision which are inherited as part of a genetic syndrome.

The association of hearing loss and cutaneous abnormalities has been previously described in a variety of mammals [31]. In humans, Waardenburg syndrome is a rare (1/40,000) autosomal dominant disorder characterized by pigmentation defects and sensorineural deafness, and eye abnormalities caused by the absence of melanocytes in the skin and the stria vascularis of the inner ear. Warburg Thomsen syndrome is an extremely rare condition characterized by cutaneous hypomelanotic and pigmented spots, microcornea, coloboma, and severe hearing loss. 

These cases likely represent a new, neurocutaneous genetic syndrome characterized by xanthomatous lesions, hearing, and vision loss. Thus, xanthoma disseminatum should be considered in the workup of a patient with hearing or vision loss who presents with cutaneous papules.
